# Working time reduction, mental health, and early retirement among part-time teachers at German upper secondary schools - a cross-sectional study

**DOI:** 10.3389/fpubh.2023.1293239

**Published:** 2023-11-23

**Authors:** Reingard Seibt, Steffi Kreuzfeld

**Affiliations:** Institute for Preventive Medicine of the Rostock University Medical Center, Rostock, Germany

**Keywords:** part-time teachers, reduced working hours, overtime, burnout, ability to recover, unpaid overtime, retirement

## Abstract

**Objective:**

Many secondary school teachers work part-time in order to cope with the high workload and to remain as healthy as possible until regular retirement. However, due to the acute shortage of teachers, the increase in the teaching obligation for part-time teachers (PTT) has become a topic of discussion in Germany. Whether a reduction in teaching hours is associated with benefits for mental health has not yet become evident. Therefore, this study investigated the relationship between the real working hours of PTT and their mental health as well as individual pension prognosis.

**Methods:**

The cross-sectional study included 5,905 PTT at German upper secondary schools (female proportion: 81%, average age: 44 ± 9 years) who logged their working hours over four weeks. Four part-time groups (PTG) were formed based on the proportion of a full-time position worked: maximum (<100–90% = PTG_max_ – reference group – 17%), high (<90–75% = PTG_high_ – 34%), medium (<75–50% = PTG_med_ – 40%) and low (<50% = PTG_low_ – 9%) teaching commitment. These groups were compared in terms of their average weekly working hours, mental health (inability to recover, risk of burnout) and predicted retirement age.

**Results:**

The contractually agreed working time is exceeded to a relevant extent for PTT. The extent of unpaid overtime increases significantly the lower the teaching obligation is and lies on average between −0.4 (PTG_max_) and 7.3 (PTG_low_) hours/week. A reduction in teaching hours is neither related to the mental health of teachers nor to their decision to retire early (42%) or regularly (58%). However, predicted retirement is mainly explained by mental health status, gender and age (variance explanation: 24%, OR of predictors: maximum 2.1). One third of PTT reported inability to recover, 47% burnout symptoms and 3% a burnout syndrome.

**Conclusion:**

Mental health is also a risk for PTT; reducing teaching hours alone does not improve it. However, good mental health increases the chance of regular retirement. Therefore, instead of a legal obligation, PTT should be encouraged to increase the number of teaching hours voluntarily in order to counteract the general shortage of teachers.

## 1 Introduction

In addition to the migration of young professionals to other professions and the taking of early retirement, the high proportion of part-time employees is also discussed as a cause of the teacher shortage in many countries within the Organization for Economic Co-operation and Development (OECD) (1). However, the demand raised by German education politicians for an increase in the teaching obligation of part-time teachers must be questioned.

The work of teachers is characterized by an extensive, varied and responsible range of activities with a substantial proportion of psychosocial interaction work (2). The resulting high emotional and mental demands of work are considered potential stressors and increase the risk of stress-related mental and psychosomatic illnesses in this occupational group, including burnout (3–6).

A particular challenge for the teaching profession is to master the complex task profile largely autonomously. On average, less than half of working time is spent on compulsory teaching hours (7, 8). All other tasks (e.g., lesson preparation, corrections, project organization) are carried out in the PTT’s own time. There are very few qualitative requirements for the fulfillment of these tasks. For some teachers, this deficit in professional orientation leads to a conflict between their professional demands and the need to protect themselves from excessively long working hours. Pedagogical ideals promote tendencies of overcommitment and a working style that is hazardous to health (9, 10). Most teachers work regularly at home, often in the evenings, weekends and on days off (11). A substantial proportion of full-time teachers at German upper secondary schools (36%) exceeds the statutory maximum working time of 48 h/week according to the European Working Time Act (7, 12).

In order to cope with the high mental, emotional and psychosocial workloads, approximately 46% of all secondary school teachers in Germany decide to work part-time. Of these, almost 80% are women (13). Part-time work is defined as any employment relationship in which fewer hours are worked than those of comparable full-time jobs (14). For teachers, the amount of part-time work varies from less than 50% to nearly 100% of the compulsory teaching hours of a full-time position, and thus individual work hours vary considerably. In this respect, part-time teachers are a very heterogeneous group.

The reasons for a reduction in teaching hours are vastly different. While some teachers hope this will result in less time pressure to complete all work-related tasks, others use part-time work to implement their ideas of good teaching (15–17). As a result, these teachers have to pay for the fulfillment of their own demands for teaching quality through unpaid extra work. This can lead to high number of weekly working hours despite part-time work.

In Germany, especially women see part-time work as an important tool to reconcile work and family life (18). Besides aspects of choosing to use time for family commitment, voluntary part-time work can also be linked to leisure orientation or self-realization. Regardless of the reasons, part-time work is typically associated with a reduction in wages, pension entitlements and career advancement opportunities (19).

There are major international differences in the extent to which teachers work part-time. While, e.g., in Switzerland the vast majority (71%) of teachers in upper secondary education worked part-time in 2020, only 1% did so in Cyprus (1). Further, earlier studies have shown that part-time teachers tend to exceed the agreed working hours (10, 20). In later, more recent representative studies, Mußmann et al. (21) for German uppers secondary school teachers and Brägger (15) for Swiss teachers confirmed that part-time teachers exceed the target working hours more than full-time teachers and interpreted this finding as an expression of an elevated level of professional commitment. In addition, it was noticed that unpaid overtime increased with a decreasing amount of teaching obligation (15). This is probably favored by the fact that there is more free time available overall.

Recovery processes seem to be an important mediator between work-related stressors and their health effects (22–25). Long working hours not only reduce recovery time, but also the possibility of mental detachment from work (26) with the result of sustained physiological activation which goes beyond working hours. Together with stress-related, cognitive processes (e.g., rumination), this hinders necessary recovery (26–29). According to a representative survey of German employees, teachers are more often unable to detach from work than other professional groups and are, after managers, the second most frequently affected by recreational impairments (30).

High job demands can also hinder detachment from work (31, 32). Although employees feel a strong need for recovery, they do not manage to recover sufficiently (33, 34). They take advantage of active recreational opportunities such as sports activities less often (35) and sleep less well (36). In the long term, this “recovery paradox” can lead to the consumption of individual performance reserves and exhaustion (37).

Occupational burnout is interpreted as physical and emotional exhaustion due to work-related problems (38). According to Maslach and Leiter (39), burnout consists of the dimensions of exhaustion, cynicism or mental distancing as well as a sense of ineffectiveness and lack of accomplishment. Thus, a considerable number of studies have dealt with the occurrence of burnout, especially among teachers (3, 6, 40–43). A divergent burnout prevalence of between 0 and 71% has also been reported (3, 40, 42), which may be due to the use of different concepts and recording methods and/or the composition of the samples.

In the teaching profession, the incidence of mental illness, including burnout, is associated with a significant risk of early retirement (44–47). The decision to leave the profession is viewed as a multidimensional process in which societal, socio-medical and individual reasons are weighed against each other (47). In Germany, only about one in four teachers reaches the statutory retirement age (48), with a sizeable proportion of teachers leaving the profession early at their own request (49). The authors’ studies on a representative sample of German full-time teachers at upper secondary schools confirm that the likelihood of leaving the profession before the regular retirement age increases when the extent of emotional exhaustion increases and the ability to recover is impaired (49).

Although numerous studies have dealt with the unfavorable effects of long working hours and high work demands on health, there is little reliable knowledge of the positive effects of reduced working hours. In a systematic review, Voglino et al. (50) reported a correlation between a reduced quality of life, sleep and stress. However, in the seven selected long-term intervention studies, only a reduction in working hours with full wage compensation has been considered, while the effects on other health indicators have remained unclear. For the group of part-time teachers, it would be interesting to know whether shorter working hours are related to better mental health. This question has not been investigated so far. Likewise, in previous studies, the significant working time differences of part-time teachers resulting from their teaching obligations have not been considered in a differentiated manner.

Therefore, this study examined groups of part-time teachers with different teaching obligations regarding their working time as well as the association with mental health (ability to recover, risk of burnout) and the predicted retirement date (early or regular). In addition, the reasons for early retirement were analyzed.

Hypothetically, it was assumed that *with decreasing teaching obligation* the following are valid:

*H Ia*: The weekly working time decreases.

*H Ib*: The amount of unpaid overtime increases.

*H II*: Mental health improves.

*H III*: The probability of remaining in the profession until the regular retirement date increases.

## 2 Methods

### 2.1 Procedure and recruitment

The data were collected as part of the Germany-wide study “Teaching Work in Transition” (LaiW study) for upper secondary school teachers between January and April 2018 (cross-sectional design). This study represented a survey of part-time upper secondary school teachers from all 16 federal states of Germany and met the representativeness requirements for the characteristics gender and age for German part-time teachers at upper secondary schools. Statistical data on the group of German school teachers beyond this is not available.

A four-week study period with an average workload was selected in each of the individual federal states in order to ensure comparable conditions for recording working hours nationwide. In preparation for the study, posters and flyers at all upper secondary schools advertised voluntary participation in the study. Before the start of the study, all teachers received a letter about the study containing information on data protection, implementation and data analysis as well as the conditions for participation and access to the study. Anonymity of the data was guaranteed using transaction numbers and an eight-digit personal code. The data was collected via an online portal of the University of Rostock.

All procedures performed were in accordance with the Helsinki declaration or comparable ethical standards. The design and all details of the study were approved by the Local Ethics Committee (A 2018-0031). Participants were informed about the study purpose, methods, and confidentiality of data. Informed consent was given by every participant prior to the inclusion in the study. The analyses were carried out in accordance with the relevant guidelines and regulations.

### 2.2 Measures

For data collection, an online protocol, (OP) and an online questionnaire (OQ) were developed and used at the Institute for Preventive Medicine of the Rostock University Medical Centre [for detailed information, see (7)]. The OP served to determine the average weekly working time (WWT) and activity structure. The OQ only had to be filled in once. Both the OP and the OQ contained input aids and default settings that prevented implausible time entries. Only participants for whom both an OP and an OQ were available were included in the data analysis. The completeness of the information in both recording methods was then checked.

#### 2.2.1 Online protocol

With the OP, the daily working hours had to be documented for four weeks (28 days) using 12 defined, teacher-specific activity categories. These were then assigned to three overarching teacher-specific work fields:

- Teaching (lessons, substitution lessons).
- Teaching-related activities (preparation and follow-up of lessons, corrections, preparation of projects and excursions).
- Non-teaching activities (work with pupils and parents, administration, work with colleagues, tasks within the scope of pupils’ inclusion and integration, supervision time, all other tasks).

For each day of instruction, the teachers had to indicate whether they were present at school and gave any lessons. In the case of absence, the appropriate reason had to be selected (personal illness, illness of relative, regular day off, other personal or official reasons, etc.).

To determine the WWT, the average values for each activity category were first calculated and then combined into the three higher-level of teacher-specific activity areas and finally into the average WWT. Weeks with absences due to illness were excluded from the calculation of the working time and the average value for the WWT was calculated from the remaining weeks. Likewise, participants who recorded their working hours for fewer than 21 days were excluded from the data analysis.

#### 2.2.2 Online questionnaire

The OQ contained questions about mental health in the form of the ability to recover and the risk of burnout as well as the probability of regular or early retirement in addition to socio-demographic information (e.g., gender, age, marital status, children, etc.) and occupation-specific information (e.g., teaching obligations, hours credited for special tasks, classes at secondary level II, further education, subjects taught, classes, number of students, etc.).

Inability to recover (IR): IR captured a habitual pattern of behavior which is associated with the ineffective use of recreational time and which impedes regeneration processes. Most items reflected characteristics of experienced work continuity that are difficult to discard (e.g., not being able to switch off) and are associated with insufficient recovery phases (51, 52).

Inability to recover is a subscale of the questionnaire for the analysis of stress-related coping requirements [FABA: (51, 52)] and was recorded with six items using a four-level Likert scale (1 = does not apply to 4 = applies considerably). The total value (IR score) was formed using these items (range: 6–24 points) and assigned to normal (6–18 points), noticeable (19–21 points) and very noticeable (22–24 points) recovery areas. A high IR score corresponded to an inability to recover and, conversely, a low IR score corresponded to good recoverability.

The reliability of the IR subscale was rated as good; Cronbach’s α was 0.79 (51). In the present study, a Cronbach Alpha of 0.79 was also determined for IR, which is on the edge of the good range (53).

Risk of burnout (RB): To assess the risk of burnout, the German translation of the Maslach Burnout Inventory – General Survey (MBI-GS) (54) was used with the three subscales emotional exhaustion (5 items), cynicism/depersonalization (5 items) reduced personal accomplishment/performance (6 items). Each item was rated on a seven-point Likert scale (0 = never to 6 = daily) according to how often it occurred.

The risk of burnout was calculated using the following formula of Kalimo et al. (55): (0.4 * emotional exhaustion) + (0.3 * cynicism) + (0.3 * reduced performance). Burnout can be suspected when emotional exhaustion and cynicism are high and performance is low. Generally, a value below 1.49 indicates no burnout symptoms, in the range from 1.50 to 3.49 points some burnout symptoms and from a point value of 3.50 a possibility of burnout syndrome (55).

Schaufeli et al. (54) reported Cronbach’s alphas of 0.87 (emotional exhaustion) to 0.64 (depersonalization; performance = 0.80) for the MBI-GS and thus good to satisfactory Cronbach’s alpha values. For the three burnout subscales in this study, Cronbach’s alpha was between 0.79 and 0.84 and therefore in the acceptable or good range (53).

Retirement date: The questions on the probability of regular versus early retirement as well as on the individual reasons were self-developed and recorded with the following global question: “Can you imagine practicing your job until the statutory (regular) retirement age?” If the question was answered with “no,” a maximum of three main reasons for the early retirement were to be given. These details were free text statements that were assigned to a self-defined category system for all teachers.

The qualitative analysis of the reasons for early retirement was based on the structuring content analysis according to Mayring (56). Thus, the category system was initially derived from preliminary theoretical considerations and then refined by two independent evaluators after reviewing and assigning the information to the provisional categories. In order to minimize the evaluators’ subjective perspective, the final categories (*n* = 12) were determined after the two evaluators had compared the allocation to the categories.

### 2.3 Data control and processing

In the run-up to the statistical calculations, the entire data set was checked for implausible information. The amount of time for the individual activity categories in the OP was examined for statistical outliers. Extreme values were replaced by subject-specific mean values in the individual activity categories. The amount of teaching and reduced teaching time was checked in the OQ based on the special tasks of the teacher and their ages.

### 2.4 Data analysis

The statistical analysis of the data was conducted using the software “Statistical Package for the Social Science” (SPSS, version 29) for Windows. Mean differences between the part-time groups were examined for the characteristics included (variables) – controlling for gender and age – using univariate covariance analyses. The Bonferroni test (post-hoc test) was added in the case of significant values of *p*. The Chi^2^-test was used for categorical variables.

To evaluate the correlations between the working time or health characteristics and the variable “part-time group” the rank correlation coefficient was calculated according to Spearman (*R*) and interpreted according to Bühl (57), for which correlation coefficients <±0.10 were considered independent of each other. The contingency coefficient (*C*) was used for nominal features. Internal consistencies were examined with Cronbach’s Alpha and evaluated according to Blanz (53).

Binary logistic regression analyses were used to investigate the influence of work and health characteristics (independent variables), including control variables, on the probability of reaching regular retirement age (response variable) (variable “retirement date” – regular versus early retirement). The assessment of the goodness of fit was based on Nagelkerke *R*^2^ (value range: 0–1) (58). The higher the Nagelkerke *R*^2^ value, the better the fit between the model and the data.

A probability of error of *α* = 5% (*p* < 0.05) was defined as a statistical significance criterion and supplemented by effect sizes. The interpretation of the effect sizes followed the conventions of Cohen (59). The respective effect sizes were calculated using the formulas of Lenhard & Lenhard (60). Practically significant results were effect sizes from *η*^2^_partial_ ≥ 0.01 for the analysis of covariance and values from *d* ≥ 0.20 for the *χ*^2^-test.

### 2.5 Sample

More than 20,000 high school teachers participated in the LaiW study. Complete OQ datasets were available for 18,791 teachers and 14,338 participants met the quality requirements in both the OP and the OQ. Part-time employment was defined as any employment in which the teaching time was less than the standard teaching obligation. Teachers who are compensated with a reduction in teaching obligation for taking on special tasks and functions (e.g., trade union tasks) were only included in the analyses if the extent of the teaching reduction did not exceed three hours per week. Thus, the participating teachers were comparable in terms of their teaching proportion of the overall working time. In total, the data of 5,905 part-time teachers were analyzed.

Since the scope of teaching among part-time teachers varied widely, they were assigned – according to the question – to the following four part-time groups: group with maximum (<100–90% = PTG_max_), high (<90–75% = PTG_high_), medium (<75–50% = PTG_med_) and low (<50% = PTG_low_) teaching obligation. The average teaching obligation of all part-time teachers was 72 ± 16% of a full-time position and was distributed among the part-time groups as follows: PTG_max_: 93 ± 2%, PTG_high_: 82 ± 4%, PTG_med_: 63 ± 8%, PTG_low_: 41 ± 6%. The composition of the sample is shown in Table 1.

**Table 1:** Characteristics of the sample.

Baseline characteristics	Part-time groups (PTG)							
	PTG_max_: <100–90%		PTG_high_: <90–75%		PTG_med_: <75–50%		PTG_low_: <50%	
	*n*	%	*n*	%	*n*	%	*n*	%
Part-time teachers	1,011	17.1	1,994	33.8	2,369	40.1	531	9.0
Gender								
Men	384	38.0	508	25.5	213	9.0	19	3.6
Women	627	62.0	1,486	74.5	2,156	91.0	512	96.4
Age groups [years]								
20–29	74	7.3	104	5.2	26	1.1	7	1.3
30–39	354	35.0	551	27.6	598	25.2	181	34.1
40–49	300	29.7	686	34.4	1,173	49.5	292	55.0
50–59	242	23.9	550	27.6	485	20.5	40	7.5
60–67	41	4.1	103	5.2	87	3.7	11	2.1
Subjects and subject combinations								
Languages	189	18.7	447	22.4	576	24.3	153	28.8
Social sciences	24	2.4	46	2.3	43	1.8	14	2.6
Natural sciences	219	21.7	379	19.0	420	17.7	101	19.0
Languages and social sciences	261	25.8	455	22.8	593	25.0	127	23.9
Languages and natural sciences	57	5.6	139	7.0	204	8.6	31	5.8
Social sciences and natural sciences	78	7.7	122	6.1	131	5.5	26	4.9
Art, music, sports	17	1.7	28	1.4	37	1.6	8	1.5
Subject combinations with art, music, sports	166	16.4	378	19.0	365	15.4	71	13.4
Family obligations								
Permanent partnership	810	80.1	1,691	84.8	2.217	93.6	512	96.4
Children in the household	382	37.8	971	48.7	1,881	79.4	497	93.6
Care of relatives	67	6.6	125	6.3	162	6.8	29	5.5

*n*, number of teachers; %, frequencies in %; PTG_max_, maximum teaching obligation; PTG_high_, high teaching obligation; PTG_med_, medium teaching obligation; PTG_low_, low teaching obligation.

17% of the teachers indicated a teaching obligation of at least 90% of a full-time position (PTG_max_); they were used in the present study as a reference group, since the associated WWT of 43.0 ± 8.4 h is considered full-time employment in other occupations. Almost three quarters (74%) of all teachers worked less than 90%, but at least 50% of the teaching obligation of a full-time position (PTG_high_, PTG_med_). For 9% of them, teaching obligation was less than half a position (PTG_low_).

Most frequently taught were languages (23%), combinations of languages and social sciences (24%), natural sciences (19%) or a combination of art, music and physical education (17%). The proportion of women of 81% corresponded to the usual distribution among part-time teachers in Germany. The vast majority taught as civil servants^1^ (86%) and 14% as employees.

At the time of the survey, the teachers were on average 44 ± 9 years old; those who worked less than 50% (PTG_low_) were on average three years younger (42 ± 6 years, *p* < 0.001, *d* = 0.322 – small effect) than those with high and medium workloads (PTG_high_, PTG_med_). Just under a third of all teachers were younger than 40 years. Most of the participants lived in a stable partnership (89%); this percentage increased slightly as teaching obligation decreased (*C* = 0.18). Almost two thirds (63%) of teachers looked after children in their own household; this applied to a good third (38%) in a position with maximum teaching load (PTG_max_), but almost all teachers (94%) in a position with low teaching load (PTG_low_) (*R* = 0.38). Only a few teachers reported having to care for other relatives in the household (6%).

## 3 Results

### 3.1 Work stress

The average values of the working times for three teacher-specific areas of activity in addition to the average weekly working time (WWT) were used to determine the weekly workload. In order to clarify the extent to which part-time teachers at German upper secondary schools perform unpaid overtime, the collectively agreed weekly TARGET working time was compared with the corresponding ACTUAL working time. The individual weekly TARGET working time was calculated based on the teaching obligation and then the difference between the TARGET and ACTUAL working time for each teacher was determined. A TARGET working time for full-time teachers of 46.4 h per week was assumed according to Mußmann et al. (61). This number of hours is considered the imputed “standard working time” during school hours. It is assumed that no work is done during vacations, public holidays and weekends.

#### 3.1.1 Weekly working time and teacher-specific activities

The results of the WWT and the working hours for the three teacher-specific areas of activity are summarized in Table 2.

**Table 2:** Main effects of weekly working time, weekly time for teacher-specific activities, and covariates (gender, age groups).

Part-time groups (PTG)	M ± SD [h/week]	*F*-value	Value of *p*	*η* ^2^_partial_
Weekly working time (WWT)				
PTG_max_: <100–90% (*n* = 1,011)	43.0 ± 8.4	612.37	<0.001***	0.238
PTG_high_: <90–75% (*n* = 1,994)	40.0 ± 8.5			
PTG_med_: <75–50% (*n* = 2,369)	33.5 ± 8.7			
PTG_low_: <50% (*n* = 531)	26.3 ± 7.8			
Gender		3.39	0.065	0.001
Age group		1.17	0.280	<0.001
Teaching [60 min]				
PTG_max_: <100–90% (*n* = 1,011)	16.1 ± 2.3	2459.65	<0.001***	0.556
PTG_high_: <90–75% (*n* = 1,994)	14.2 ± 2.2			
PTG_med_: <75–50% (*n* = 2,369)	11.0 ± 2.0			
PTG_low_: <50% (*n* = 531)	7.6 ± 1.9			
Gender		9.33	0.002**	0.002
Age group		26.62	<0.001***	0.004
Teaching related activities				
PTG_max_: <100–90% (*n* = 1,011)	18.3 ± 7.5	85.25	<0.001***	0.042
PTG_high_: <90–75% (*n* = 1,994)	17.4 ± 7.3			
PTG_med_: <75–50% (*n* = 2,369)	15.4 ± 6.9			
PTG_low_: <50% (*n* = 531)	12.9 ± 6.2			
Gender		7.85	0.005**	0.001
Age group		1.08	0.300	<0.001
Non-teaching activities				
PTG_max_: <100–90% (*n* = 1,011)	8.6 ± 4.4	102.38	<0.001***	0.049
PTG_high_: <90–75% (*n* = 1,994)	8.4 ± 4.4			
PTG_med_: <75–50% (*n* = 2,369)	7.1 ± 4.1			
PTG_low_: <50% (*n* = 531)	5.7 ± 4.0			
Gender		46.30	<0.001***	0.008
Age group		4.58	0.032*	0.001

PTG_max_, maximum teaching obligation; PTG_high_, high teaching obligation; PTG_med_, medium teaching obligation; PTG_low_, low teaching obligation; M ± SD, means ± standard deviations. General linear model, internal subject design: constant term + gender + age group; test variable: *F*-value; df = 2; error def = 5,905; value of *p*, significance (two-sided): ***p < 0.001, **p < 0.01, *p < 0.05; *η*^2^_partial_, partial eta square (effect size): <0.010 = no effect, 0.010–0.059 = small effect, 0.060–0.139 = medium effect, ≥0.140 = large effect (59). Corrected *R*^2^: weekly working time = 0.258, teaching = 0.583, teaching related activities = 0.051, non-teaching activities = 0.049.

For the total sample of part-time teachers, the average WWT is 37 hours. As expected, the average WWT differs significantly between the part-time groups (*η*^2^_partial_ = 0.238 – large effect). While the teachers in the PTG_max_ work an average of 43 h/week, this working time in PTG_low_ is only 26 h/week. However, the WWT also varies greatly in the part-time groups. The differences in teaching obligation explain 26% of the differences in weekly working hours (*R* = −0.51). On average, part-time teachers teach 17 (SD ±5) school hours (45 min each) per week, which corresponds to a WWT of 13 hours. The teaching obligation in PTG_max_ is an average of 21 school hours and in the PTG_low_ an average of only 10 school h/week (*η*^2^_partial_ = 0.556 – large effect).

Teaching-related activities require an average WWT of 16 h for part-time teachers, non-teaching activities eight hours. These activities also decrease with decreasing teaching-related activities: *R* = −0.21, non-teaching activities: *R* = −0.19. The spectrum ranges from an average of 18 to 13 h/week for teaching-related activities (*η*^2^_partial_ = 0.042 – small effect) and from an average of nine to six h/week for non-teaching activities (*η*^2^_partial_ = 0.049 – small effect). The amount of time for non-teaching activities does not differ between PTG_max_ and PTG_high_ (*p* = 0.831); both part-time groups invest an average of eight hours of their WWT for this. Overall, activities close to and distant from lesson activities explain only 5% of the lesson obligation each (*R* = −0.23).

Viewed in a differentiated manner, teachers spend an average of 9.5 ± 4.2 h/week in PTG_max_ and 7.2 ± 3.2 h/week in PTG_low_ for lesson preparation and follow-up (PTG_high_: 9.2 ± 4.2 h/week, PTG_med_: 48.2 ± 7.3 h/week, *F*(3) = 63.55, *p* = 0.001, *η*^2^_partial_ = 0.031). The WWT for corrections varies on average between 6.2 ± 4.2 (PTG_max_) and 4.4 ± 3.5 h/week (PTG_low_) (PTG_high_: 5.9 ± 4.2 h/week, PTG_med_: 5.4 ± 3.5 h/week, *F*(3) = 31.72, *p* = 0.001, *η*^2^_partial_ = 0.016). Gender and age have no significant influence on the teacher-specific occupations and consequently neither on the average WWT (*η*^2^_partial_ < 0.010).

**Figure 1:**
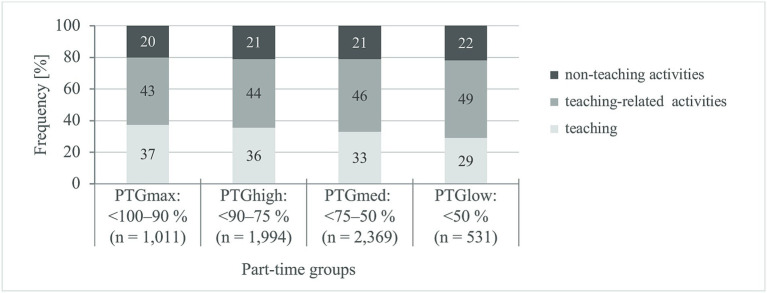
Teacher-specific activities on the weekly total working time. PTG_max_, maximum teaching obligation; PTG_high_, high teaching obligation; PTG_med_, medium teaching obligation; PTG_low_, low teaching obligation. General linear model, internal subject design: constant term + gender + age group; test variable: *F*-value; df = 3; error def = 5,905. Teaching: *F* = 91, *p* < 0.001, *η*^2^_partial_ = 0.044, teaching related activities: *F* = 37, *p* < 0.001, *η*^2^_partial_ = 0.019, non-teaching activities: *F* = 9, *p* < 0.001, *η*^2^_partial_ = 0.004. *η*^2^_partial_, partial eta square (effect size): <0.010 = no effect, 0.010–0.059 = small effect (59).

Considering the three teacher-specific areas of activity as a percentage of the WWT, the following becomes clear (Figure 1): pure teaching covers an average of 35% (SD ±10) of the working time of part-time teachers. It averages 37% at maximum teaching obligation and decreases to 29% with decreasing teaching obligation. On average, teaching-related activities take up 44% (SD ±11) and non-teaching activities 21% (SD ±9) of the WWT. Significantly, the proportion of teaching-related activities increases with decreasing teaching obligation, whereas for non-teaching activities it is comparable in the part-time groups.

#### 3.1.2 Comparison of TARGET and ACTUAL weekly working time

**Figure 2:**
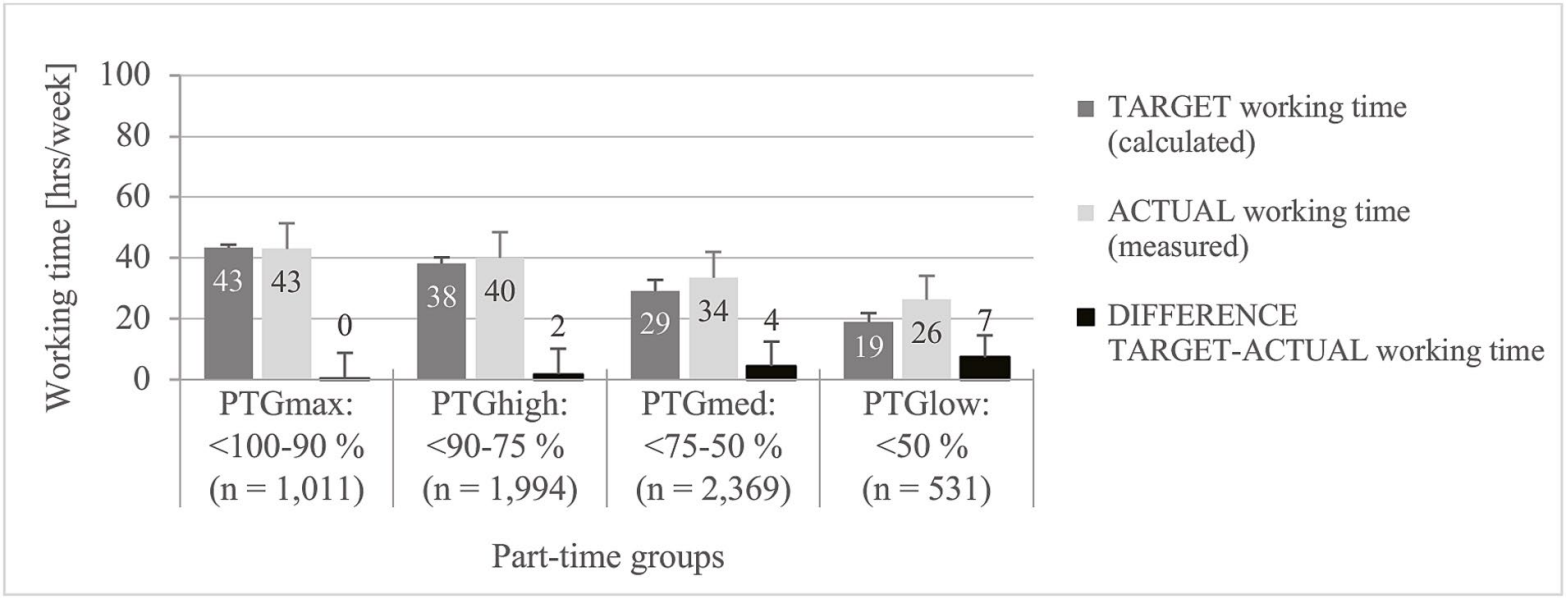
Comparison of calculated and measured weekly working time. PTG_max_, maximum teaching obligation; PTG_high_, high teaching obligation; PTG_med_, medium teaching obligation; PTG_low_, low teaching obligation. Means ± standard deviations. General linear model, internal subject design: constant term + gender + age group; test variable: *F*-value; df = 3; error def = 5,905. TARGET working time (calculated): *F* = 13,320, *p* < 0.001, *η*^2^_partial_ = 0.871, difference TARGET-ACTUAL working time: *F* = 133, *p* < 0.001, *η*^2^_partial_ = 0.064. *η*^2^_partial_, partial eta square (effect size): 0.060–0.139 = medium effect, ≥0.140 = large effect (59). Corrected *R*^2^: TARGET working time (h/week) = 0.872, difference TARGET-ACTUAL working time (h/week) = 0.068.

The results of the TARGET-ACTUAL working hours on the basis on a full-time position (46.4 h/week) are summarized in Figure 2 according to Mußmann et al. (61).

The TARGET-ACTUAL differences in weekly working hours confirm that the overtime performed increases significantly with decreasing teaching obligation [*F*(3) = 133, *p* < 0.001, *η*^2^_partial_ = 0.064 – average effect]. With maximum teaching obligation (PTG_max_), the average WWT would be around 43 h, and with low teaching obligation (PTG_low_) only 19 hours. While the logged ACTUAL working time in PTG_max_ is on average even 0.4 h below the TARGET working time, an average of seven hours of unpaid overtime/week were recorded for PTG_low_. However, the standard deviations make it clear that some part-time teachers work even less than the TARGET working hours.

The “standard working time” for full-time teachers of 46.4 h/week (61) is exceeded by 15% of all part-time teachers, whereby the percentage of the excess decreases with decreasing teaching obligation (PTG_max_: 30%, PTG_high_: 20%, PTG_med_: 8%, PTG_low_: 2%, *d* = 0.525 – large effect). Gender and age had no significant influence on this result (*p* > 0.05).

In summary, the results confirm hypotheses Ia and Ib: as the amount of teaching decreases, the total weekly working time also decreases; however, the unpaid overtime increases.

### 3.2 Mental health

With regard to mental health, it is necessary to investigate whether a reduced teaching obligation leads to a better ability to recover (52) and a lower risk of burnout (55).

#### 3.2.1 Inability to recover (IR)

**Figure 3:**
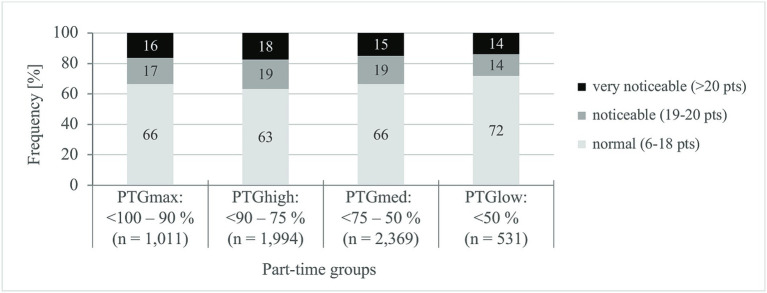
Specifications of inability to recover. PTG_max_, maximum teaching obligation; PTG_high_, high teaching obligation; PTG_med_, medium teaching obligation; PTG_low_, low teaching obligation; pts, points. *χ*^2^-test (Pearson), *χ*^2^, test size. *χ*^2^(6) = 16, *p* = 0.013, *d* = 0.052. Value of *p*, significance (two sided): *p* < 0.05; *d*, effect size: <0.100 = no effect (59). Corrected *R*^2^ = 0.019.

The results on the inability to recover are visible for the part-time groups in Figure 3.

On average, the part-time groups differ significantly in their ability to recover [*F*(3) = 13, *p* < 0.001, *η*^2^_partial_ = 0.007]. However, the differences are practically irrelevant. The mean value of the IR score in the total sample with 17 points (SD ±4) can still be classified as normal (51, 52), but is close to the limit of the abnormal range (>18 points). Irrespective of this, about a third of the part-time teachers (34%) report insufficient recovery in their free time.

The ability to recover is only relevantly influenced by gender [*F*(1) = 83, *p* < 0.001, *η*^2^_partial_ = 0.014 – small effect], according to which women (IR: 36%) are more frequently affected by the inability to recover than men (IR: 26%). Age has no significant effect on recovery ability [*F*(1) = 11, *p* = 0.001, *η*^2^_partial_ = 0.002].

#### 3.2.2 Risk of burnout

**Figure 4:**
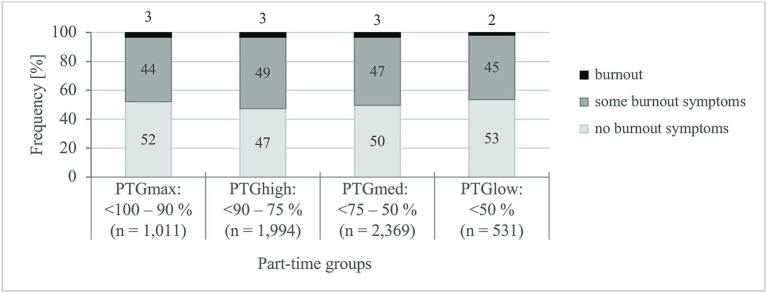
Specifications of risk of burnout. PTG_max_, maximum teaching obligation; PTG_high_, high teaching obligation; PTG_med_, medium teaching obligation; PTG_low_, low teaching obligation. *χ*^2^-test (Pearson), *χ*^2^, test size. *χ*^2^(6) = 13, *p* = 0.044, *d* = 0.094. Value of *p*, significance (two-sided): *p* < 0.05; *d*, effect size: <0.200 = no effect (59).

The findings on the risk of burnout (55) for the part-time groups are compared in Figure 4. The average risk of burnout (range: 0–6 points) is reported as 1.7 points (SD ±0.9) and differs only slightly between the part-time groups (points: PTG_max_: 1.6 ± 0.8, PTG_high_: 1.7 ± 0.9, PTG_med_: 1.6 ± 0.9, PTG_low_: 1.5 ± 0.8, *F*(3) = 4, *p* = 0.007, *η*^2^_partial_ = 0.002). The differences are also practically insignificant. Gender [*F*(1) < 1, *p* = 0.438] and age effects [*F*(1) < 1, *p* = 0.421] do not exist for the risk of burnout.

According to the assessment of the risk of burnout (55), half of all part-time teachers have no symptoms of burnout, 47% of them have some symptoms of burnout and 3% have evidence of burnout syndrome. There is no correlation to be found between the risk of burnout and the extent of the teaching obligation (*R* < ±0.10).

In summary, Hypothesis II must be rejected: the part-time groups do not differ in terms of their mental health (ability to recover and risk of burnout).

### 3.3 Date of retirement

**Figure 5:**
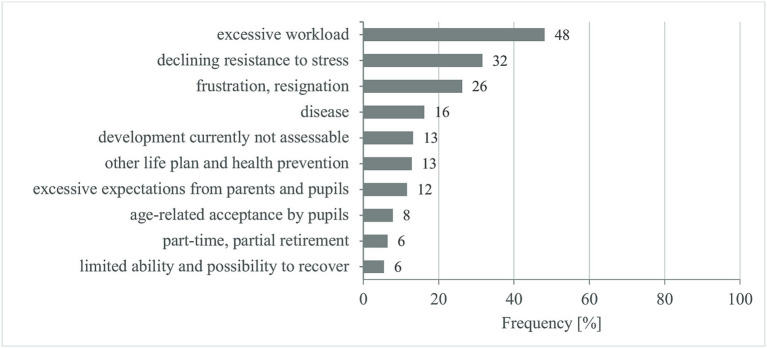
Reasons for early retirement (multiple responses possible).

The results on the relation between working time reduction and retirement are reported in the following. The reasons given for early retirement are summarized in Figure 5. The extent of the teaching obligation has no significant influence on whether part-time teachers want to retire early (*p* < 0.001, *d* = 0.171 – no effect). Overall, 42% of part-time teachers intend to retire early. This proportion is largest in PTG_high_ (46%) and, as expected, smallest in PTG_low_ (31%) (PTG_max_: 38%, PTG_med_: 44%). In addition, more women (45%) than men (32%) intend to retire early.

Since the reasons for early retirement do not differ between the part-time groups (*p* = 0.632), they are reported for the entire group of part-time teachers (Figure 5). Overall, 4% of the part-time teachers surveyed gave no reason and 41% gave only one; more than a third (35%) gave two and just under a quarter (24%) three reasons for early retirement.

Almost half (48%) of the part-time teachers state excessive workload as the main reason for early retirement. About a third (32%) sees no possibility of working until the regular start of retirement due to age-related declining mental and physical performance and resilience. And still more than a quarter (26%) are so frustrated because of the general school conditions (e.g., bureaucracy, requirements of the Ministry of Education, classes too large and too heterogeneous, bad working conditions, hopelessness in terms of improvements) that they would like to leave teaching as soon as possible. This also includes the reasons that address a limited ability and opportunity to recover (6%).

Reasons for illness (e.g., emotional exhaustion, voice problems) are given by 16% of the part-time teachers; 12% have a different life plan (e.g., professional re-orientation, more free time, partner is older and is more likely to retire) and would like to enjoy their retirement with their partners in good health for as long as possible. For another 12%, reasons to seek early retirement are the excessive demands of parents and pupils, but also increasing problems with discipline and pupils’ lack of motivation. Another 8% see increasing acceptance problems among students for older teachers (e.g., age discrimination, excessive age difference between teachers and students); they do not wish to be seen as “old” by the pupils. On the other hand, 6% of part-time teachers can imagine working until the normal retirement age if the number of teaching hours is further reduced or partial retirement is possible. Younger teachers (13%) make early retirement dependent on working conditions and their state of health. For a few women (1%), caring for relatives is also a reason for early retirement.

### 3.4 Relationships between work stress, mental health, and retirement date

First, the relationship between working time and health characteristics and retirement age (regular versus early) was examined using the Eta coefficient. According to this, there are no correlations with working time characteristics (*Eta* = 0.01–0.07) and low correlations with the characteristics of mental health (Eta: IR = 0.30, RB = 0.34), i.e., the better the mental health, the better the chance of reaching normal retirement age. Gender (*C* = −0.11) and (current) age also correlate only very slightly (*Eta* = −0.17) with the planned age of retirement.

In the second step, a binary logistic regression model was used to estimate the probability by which working time and health characteristics as well as covariates determine early retirement. Since the selection of the characteristics that went into the overall model (method: enter) is based on the results of the preceding correlation analysis, only the health characteristics (here: IR, RB), control variables (here: gender, age) and the variable “part-time group” were entered into the overall model (Table 3).

**Table 3:** Binary logistic regression model of health characteristics, covariates, and scope of employment of part-time teachers reaching regular versus early retirement.

Total model	Coefficient (B)	Standard error of (B)	Wald statistic	Value of *p*	Estimated odds ratio	Confidence interval for Exp (B)	
					Exp (B)	Lower limit	Upper limit
Inability to recover [pts]	0.112	0.01	127.83	<0.001***	1.118	1.097	1.140
Risk of burnout [pts]	0.751	0.04	336.19	<0.001***	2.119	1.956	2.296
Gender (Women = 0, Men = 1)	0.630	0.09	59.63	<0.001***	1.877	1.600	2.203
Age [years]	0.042	0.01	148.52	<0.001***	1.043	1.036	1.050
Part-time groups							
PTG_max_: <100–90% (*n* = 1,011)			23.08	<0.001***			
PTG_high_: <90–75% (*n* = 1,994)	0.103	0.09	1.38	0.240	1.109	0.933	1.317
PTG_med_: <75–50% (*n* = 2,369)	−0.039	0.09	0.19	0.660	0.962	0.810	1.143
PTG_low_: <50% (*n* = 531)	−0.445	0.13	12.28	<0.001***	0.641	0.500	0.822
Constant	−5.832	0.24	587.41	<0.001***	0.003		

Dependent variable: regular versus early retirement = 0–1 coded; teaching obligation: reference category (0) = PTG_max_, maximum teaching obligation (reference group); PTG_high_, high teaching obligation; PTG_med_, medium teaching obligation; PTG_low_, low teaching obligation. Binary logistic regression (method: enter), Exp (B), expected ß. *CI*, confidence interval. Value of *p*, significance (two-sided): ***p < 0.001. Nagelkerke *R*^2^:=0.237.

With this model, a variance explanation of 24% (Nagelkerke *R*^2^ = 0.237) is achieved (Table 3), which is considered an acceptable fit between the overall model and the data (goodness of fit) (58, 62). However, the model only correctly predicts part-time teachers who plan to retire early (1,367 out of 2,493) in 55% of cases, while 81% of part-time teachers who plan to retire regularly are assigned correctly. Overall, 70% of all cases are correctly predicted.

All model variables show a significant (*p* < 0.001) influence on retirement age. However, the likelihood of early retirement only decreases when teaching obligations are less than 50% of a full-time position (PTG_low_).

With a differentiated consideration of the independent characteristics, the control variables and the variable “part-time group” contribute 6% to the explanation of early retirement. The variable burnout risk (16%) provides the highest explanation of an individual characteristic. Together with the ability to recover, both health variables explain 19% of the likelihood of early retirement. According to Chen et al. (63) however, these are only weak effects overall (*OR*: 1.50–2.99).

In summary, hypothesis III can be partially confirmed.

## 4 Discussion

The shortage of teachers is not only an educational policy problem in Germany, but also poses challenges to many other OECD countries (46, 64). In Germany, e.g., discussions are being held about closing the supply gap by increasing the number of hours taught by part-time teachers. At first glance, this measure appears to be efficient because part-time work is widespread in education. However, it is mainly carried out by female teachers (13). In addition to family reasons, stress-related and health reasons are also given for the decision to work part-time.

The individual working hours of part-time teachers vary greatly, depending on the teaching obligation set by the collective agreement. The consideration of average working hours of part-time teachers is not very meaningful. Therefore, four groups of part-time teachers with different teaching obligations were compared in this study, which confirms the findings of earlier studies (15, 21, 65) that part-time teachers at upper secondary schools perform unpaid overtime to a relevant extent.

The scope of this additional work increases the lower the collectively agreed teaching obligation is (*η*^2^_partial_ = 0.064). This applies equally to teachers and all age groups (*η*^2^_partial_ 0.010). Contrary to expectations, however, the extent of the teaching obligation is not related to the characteristics of mental health examined (ability to recover, risk of burnout) and has no influence on the decision as to whether a teacher retires early or not. Rather, this decision is explained by the status of mental health, gender and age (explained variance: 24%), with only weak effects resulting for the individual predictors (*OR*: 1.0–2.2).

As expected, the average working time/week differs significantly between the part-time groups (*η*^2^_partial_ = 0.238); it varies between 43 (PTG_max_) and 26 h/week (PTG_low_), but explains only 26% of the variation in weekly working hours. In addition, the high standard deviations of eight to nine h/week in each part-time group indicate large inter-individual differences in working hours. For the group with the highest teaching obligation (PTG_max_), this means that almost a quarter (24%) of these part-time teachers exceed the European working time limit of 48 h/week. Even in the part-time group with a teaching obligation of 50 to <75% of a full-time position (PTG_med_) and an average working time of 34 ± 9 h/week, 6% of the part-time teachers are still above this Europe-wide working time limit. Compared to other sectors, the recorded working times of the teachers in the part-time groups with a teaching obligation of more than 75% (PTG_max_, PTG_high_) fall within the range of full-time work.

Based on the calculated TARGET working times for part-time teachers, there is on average no overtime in the group with the highest teaching obligation, while part-time teachers with less than 50% teaching obligation use an average of seven h/week of their free time to cope with work tasks.

The differentiated analysis of the work tasks shows that the overtime in the area of teaching-related activities is mainly for lesson preparation and follow-up. For example, teachers with less than 50% teaching obligation spend almost half their working hours (49%) on teaching-related activities, which is significantly more than teachers with a maximum teaching obligation (43%). In contrast, the proportion of non-teaching activities is significantly lower in all part-time groups (20–22%) and comparable in scope; since participation in school conferences, parents’ days, excursions, school festivals and other events requires the presence of all teachers. It is noteworthy that the proportion of time devoted to teaching – the main task of teachers – is reduced to less than a third of the working time when teachers greatly reduce the scope of their teaching obligation (PTG_low_: 29% vs. PTG_max_: 37%, *η*^2^_partial_ = 0.556).

It is known from research that the time required for lesson preparation and follow-up as well as for corrections is significantly determined by the subjects taught (66, 67). Therefore, teachers in correction-intensive subjects (above all languages) see the need to reduce their teaching obligation. The present study confirms that there are significantly more teachers in the group with the lowest teaching obligation (PTG_low_) who teach languages (29%) than in the group with the greatest teaching obligation (PTG_max_: 19%).

In summary, part-time teachers, on the one hand, do a significant amount of unpaid overtime, presumably because they want to perform their duties to high professional quality standards. Gicheva (68) emphasized altruism and a pro-social attitude as important reasons for regular unpaid overtime work among teachers. On the other hand, they give up a significant part of their salary and their future pension and accept poorer career opportunities. It would therefore be correct not to speak of “part-time teachers,” but to take a more differentiated look at teaching obligation and actual working hours.

Maintaining mental health requires teachers to develop a healthy distance from their professional demands. Following the stressor detachment model (26), it has been assumed that part-time teachers are better able to recover than full-time ones because they are less exposed to professional stressors and have more time available for recovery. Schiller et al. (69) were able to prove that for full-time teachers (75% women) a decrease in weekly working hours of 25% with full wage compensation not only reduced the professional but also the family burden by one hour a day and free time was almost completely used for recreational activities. They also showed that time spent with housework and children supports detachment from work and thus promotes subsequent recovery processes.

In the study presented, the part-time groups do not differ significantly in terms of their ability to recover or the risk of burnout (*η*^2^_partial_ < 0.010), despite different workloads. Good mental health can be assumed for 40% of part-time teachers. They are capable of recovery and show no signs of being at risk of burnout. However, every third part-time teacher (34%) considers the amount of leisure time to be insufficient, with women (36%) reporting an inability to recover significantly more often than men (26%) (*η*^2^_partial_ = 0.014). In some studies, the inability to recover has been highlighted as an independent risk factor for reduced mental health (24, 25).

Overall, the mental health of a significant proportion of the teachers surveyed is at risk, since almost half of the teachers (47%) indicate “some burnout symptoms” and for 3% there are indications of a “burnout syndrome.” A quarter of all part-time teachers are even conspicuous due to their inability to recover and their simultaneous burnout symptoms. This finding is worrying since emotional exhaustion, a core component of burnout, is positively associated with intention to leave the job (45, 46).

Overall, the lower workload for part-time teachers does not appear to result in a health benefit. This finding is consistent with results from earlier teacher studies by Unterbrink et al. (70) and is currently supported by studies by Bodendieck et al. (71) and Du Bois et al. (72), in which the relation between reduced working hours and the risk of burnout was examined in other occupational groups. From this it can be deduced that the protection of mental health and in particular the prevention of burnout requires more than a reduction in working hours. However, according to the results of a systematic review by Voglino et al. (50), reducing working hours with the same salary is an effective measure to improve the well-being of employees and has a particularly positive effect on stress perception and sleep. Buhl and Acosta (73) concluded that reducing working hours improves well-being, especially if it leads to more time for enjoyable activities. A recent study by Neubert et al. (74) was able to prove positive effects on individual well-being even despite a reduced income. Employees with a lower weekly working time reported both higher life satisfaction and fewer symptoms of burnout.

In the present study, the risk of burnout is not influenced by gender and age. However, data on this are inconsistent. Studies by Wang et al. (75) and Arvidsson et al. (40) showed a higher degree of emotional fatigue for female teachers as opposed to male teachers. This finding is justified by the higher burden on women due to their family obligations and the conflict between family and work (75, 76). For teachers in this study it was noted that the number of children cared for in their own household increases significantly with decreasing teaching obligation (Table 1). As a result, teachers may not be able to effectively use their free time outside of work for their own recreation. Reducing leisure activities and sleeping times can in turn lead to increased tiredness and increase the need for rest.

Findings from the Sixth European Working Conditions Survey show that caring responsibilities and unpaid housework are still unequally distributed between women and men (77). In the Czech teacher study by Ptáček et al. (78), however, the authors point out that time spent with family can reduce the risk of burnout.

Mental illness is closely linked to the risk of early retirement (46). Some teachers only become aware of the finiteness of their own resources when they are emotionally exhausted or already suffering from burnout syndrome. At this point at the latest, there is a real danger that they will have to retire early. Following Harris & Adams (79), the proportion of teachers who retire early is comparable to that in other social professions (e.g., nurses, social workers). However, teachers retire much earlier than employees in other social professions. Whether the high work demands in the teaching profession actually pose a health risk and increase the risk of burnout depends primarily on the working conditions and how they are dealt with personally (80).

So far, scant attention has been paid to the specific determinants of early retirement among teachers. However, there is consensus that early retirement is a multidimensional process influenced by a variety of individual health, family, financial and work-related factors (47, 81–83). In the study presented here, 42% of all part-time teachers intend to leave the profession before regular retirement; this affects around a third (36%) of female teachers, but only 6% of male teachers. Also, in the Belgian study by Van Droogenbroeck and Spruyt (47), more female teachers than male teachers wanted to retire early and part-time teachers earlier than full-time teachers. Emotional exhaustion and dissatisfaction with extracurricular activities were other reasons for deciding to take early retirement.

In the regression model of this study, the characteristics of mental health (risk of burnout, inability to recover), gender and, in addition, age were identified as predictors of retirement. The explanation of the variance of the model suggests that retirement is not only influenced by these predictors but also by other characteristics not investigated here. Overall, only 70% of all cases are correctly forecast. Unexpectedly, reducing teaching hours does not seem to affect the decision to retire, although 48% of the part-time teachers surveyed cited excessive workload as the main reason for early retirement. Although this information does not differ significantly between the part-time groups, it is striking that this main reason was given by both 44% of the part-time teachers with the highest teaching obligation (PTG_max_) and half of the teachers with a teaching obligation below 50% (PTG_low_).

Overall, the study presented here does not find any evidence of a correlation between reduced teaching obligations and mental health for part-time secondary school teachers. A higher chance of reaching the regular retirement age is assumed only for these teachers who teach less than 50% of a full-time position. In the context of the extent of teaching obligation, the results support the thesis that part-time teachers tend to “self-exploit” themselves. Thus, longitudinal studies with well-defined samples are needed to better understand the effects of reduced teaching obligation on health.

### 4.1 Specifics of the study

The special feature of this study is that, for the first time, data on the working hours and mental health status of part-time teachers are presented from the nationwide secondary school sample, taking into account the main influencing factors. The sample distinguishes itself from other teacher studies by the differentiated consideration of part-time groups with a defined difference in teaching obligation. It is sufficiently large and fairly representative with respect to gender, and age for German part-time teachers at upper secondary schools. In addition, mixing teachers with officials (e.g., staff councils) and managers was consistently avoided.

When determining the working hours of the teachers, it should be emphasized that these hours were recorded daily over a period of four weeks with 12 defined activity categories. Although this is only an excerpt from one school year, the period covered can be regarded as representative of the average teaching time/year. The questions on the individual reasons for early retirement were free text answers (maximum three reasons) and were evaluated and categorized manually for all part-time teachers.

### 4.2 Limitations of the study

For reasons of time and economy, but also for reasons of reasonableness, it was possible to examine only part of the network of relationships between workload and health among upper secondary school teachers in the present study. In-depth analyses of private areas of life and their relations to work (e.g., work-life balance/conflict) are missing.

A random selection cannot be fully guaranteed due to the voluntary nature of the participation. It should also be emphasized that the cross-sectional design of this study does not allow a causal interpretation of the regression-analytical relationships between the examined characteristics and the predicted retirement age of the teachers.

It is critically noted that the description of teachers’ mental health in the present study was limited only to the constructs of recovery ability and risk of burnout. This leaves out other facets and psychosocial models that would have allowed for a more comprehensive view of teachers’ mental health. The effort-reward imbalance model was referred to in a previous paper (24).

Since the risk of burnout was self-reported, it cannot be concluded that the severity of the burnout syndrome is clinically relevant. Overall, the known restrictions for questionnaires apply to data collection.

The logging of working hours represented an additional time burden for the teachers, which may have had an impact on the recruitment of participants. It is possible that teachers with heavy professional and private workloads did not take part in the study due to a lack of time. In addition, the image of the population is characterized by the “healthy worker effect,” so that health risks may have been underestimated.

The activity categories for recording working hours are based on previous teacher studies (65). They represent a compromise between accuracy and practicality for time recording.

### 4.3 Conclusions for school practice and outlook

Teacher shortages are currently an unresolved issue in education policy. In order to counteract the mismatch between supply and demand in the teaching profession, increasing the teaching obligation for part-time teachers is being discussed as a potential measure. The results here confirm that unpaid overtime work can be regarded as guaranteed for part-time teachers. Since there is no link between reduced working hours and mental health, it is assumed that teachers have individually adjusted their teaching obligation to maintain their mental health. From this perspective, a legally obligatory increase in teaching hours for part-time teachers must be viewed critically. Moreover, an increase in the obligation to teach should be promoted on a voluntary basis, e.g., when the teachers’ own children have reached adolescence. In its 2005 report “Teachers Matter,” the OECD proposed offering teachers more flexible forms of employment in order to keep them in their jobs (84).

Occupational health findings and the results of this study (34% inability to recover, 47% burnout symptoms, 3% burnout) underline that work-related stress in the teaching profession can be hazardous to health and result in a risk of disability due to mental illness. For this reason alone, the workload of teachers should be organized and designed in such a way that overtime is avoided and sufficient rest is guaranteed. To this end, various relief measures (e.g., reducing extracurricular activities) should be combined with occupational health and safety and coordinated with one another. This includes professional occupational medical and psychological support that includes early indicators of health risks in order to identify endangered teachers in good time and determine the need for action. The aim must be to counteract the widespread early retirement of teachers. The recording of the inability to recover can serve as an early indicator of mental health (24).

## Data availability statement

The datasets presented in this article are not readily available because of data protection obligations toward participants. Requests to access the datasets should be directed to steffi.kreuzfeld@uni-rostock.de.

## Ethics statement

The studies involving humans were approved by Ethics Committee of the University of Rostock (A 2018-0031). The studies were conducted in accordance with the local legislation and institutional requirements. The participants provided their written informed consent to participate in this study.

## Author contributions

RS: Conceptualization, Data curation, Formal analysis, Funding acquisition, Investigation, Methodology, Resources, Validation, Visualization, Writing – original draft, Writing – review & editing. SK: Conceptualization, Data curation, Funding acquisition, Investigation, Methodology, Project administration, Resources, Validation, Writing – original draft, Writing – review & editing.

## Footnotes

^1^In the German state education system, teachers who have the status of *Beamte* are civil servants with tenured positions, affording them certain rights and privileges. All other state-employed teachers are employees and thus untenured.

## References

[1] Organization of Economic Co-operation and Development. (2020). TALIS 2018 Results (Volume II): Teachers and school leaders as valued professionals. Available at: https://www.oecd.org/education/talis-2018-results-volume-ii-19cf08df-en.htm (Accessed July 13, 2023)

[2] Skaalvik EM Skaalvik S. Job demands and job resources as predictors of teacher motivation and well-being. Soc Psychol Educ. (2018) 21:1251–75. doi: 10.1007/s11218-018-9464-8

[3] García-Carmona M Marín MD Aguayo R. Burnout syndrome in secondary school teachers: a systematic review and meta-analysis. Soc Psychol Educ. (2019) 22:189–208. doi: 10.1007/s11218-018-9471-9

[4] Guglielmi RS Tatrow K. Occupational stress, burnout, and health in teachers: a methodological and theoretical analysis. Rev Educ Res. (1998) 68:61–99. doi: 10.2307/1170690

[5] Shirom A Oliver A Ehdoa S. Teachers’ stressors and strains: a longitudinal study of their relationships. Int J Stress Manag. (2009) 16:312–32. doi: 10.1037/a0016842

[6] Skaalvik EM Skaalvik S. Dimensions of teacher burnout: relations with potential stressors at school. Soc Psychol Educ. (2017) 20:775–90. doi: 10.1007/s11218-017-9391-0

[7] Kreuzfeld S Felsing C Seibt R. Teachers’ working time as a risk factor for their mental health – findings from a cross-sectional study at German upper-level secondary schools. BMC Public Health. (2022) 22:307. doi: 10.1186/s12889-022-12680-5, PMID: 35164735 PMC8845294

[8] Organization of Economic Co-operation and Development. (2019). Education at a Glance. Available at: https://www.oecd-ilibrary.org/content/publication/f8d7880d-en (Accessed July 13, 2023)

[9] Baeriswyl S Bratoljic C Krause A. How homeroom teachers cope with high demands: effect of prolonging working hours on emotional exhaustion. J School Psychol. (2021) 85:125–39. doi: 10.1016/j.jsp.2021.02.002 33715777

[10] Hübner P Werle M. Arbeitszeit und Belastung Berliner Lehrerinnen und Lehrer In: Buchen S Carle U Döbrich P Hoyer H-D Schönwälder H-G, editors. Jahrbuch für Lehrerforschung. Weinheim, München: Juventa (1997). 203–26.

[11] Felsing C Kreuzfeld S Stoll R Seibt R. App-basierte vs. geschätzte Ermittlung der Arbeitszeit von Gymnasiallehrkräften. Präv Gesundheitsf. (2019) 14:281–9. doi: 10.1007/s11553-018-0682-x

[12] European Union. (2023). Working hours. Available at: https://europa.eu/youreurope/business/human-resources/working-hours-holiday-leave/working-hours/index_en.htm (Accessed July 13, 2023)

[13] Statistisches Bundesamt. (2021). Bildung und Kultur. Allgemeinbildende Schulen, Schuljahr 2020/2021. Available at: https://www.destatis.de/DE/Themen/Gesellschaft-Umwelt/Bildung-Forschung-Kultur/Schulen/Publikationen/Downloads-Schulen/statistischer-bericht-allgemeinbildende-schulen-2110100227005.html (Accessed August 20, 2023)

[14] International Labour Organization. (1994). TIlo. C175 – Part-Time Work Convention. Available at: https://www.ilo.org/dyn/normlex/en/f?p=NORMLEXPUB:12100:0::NO::P12100_ILO_CODE:C175 (Accessed July 13, 2023)

[15] Brägger M Schwendimann B. Entwicklung der Arbeitszeitbelastung von Lehrpersonen in der Deutschschweiz in den letzten 10 Jahren. Präv Gesundheitsf. (2022) 17:13–26. doi: 10.1007/s11553-021-00835-y

[16] Cau-Bareille D Teiger C Volkoff S. Revealing the hidden processes behind discrimination against part-time teachers in France: a lever for improving their situation In: Bagnara S Tartaglia R Albolino S Alexander T Fujita Y, editors. Proceedings of the 20th congress of the international ergonomics association 2018. Advances in intelligent systems and computing. Cham: Springer (2019). 259–68.

[17] Gehrmann A. Der professionelle Lehrer: Muster der Begründung - empirische Rekonstruktion. Opladen: Leske Budrich (2003). p. 323–324.

[18] Wanger S. (2015). Traditionelle Erwerbs- und Arbeitszeitmuster sind nach wie vor verbreitet. Institut für Arbeitsmarkt- und Berufsforschung (IAB) der Bundesagentur für Arbeit. Available at: https://doku.iab.de/kurzber/2015/kb0415.pdf (Accessed August 20, 2023)

[19] Bell LA Freeman RB. The incentive for working hard: explaining hours worked differences in the US and Germany. Labour Econ. (2001) 8:181–202. doi: 10.1016/S0927-5371(01)00030-6

[20] Schönwälder H-G Berndt J Ströver F Tiesler G. Belastung und Beanspruchung von Lehrerinnen und Lehrern. Bremerhaven: Wirtschaftsverlag NW (2003). p. 214–218.

[21] Mußmann F. Die Arbeitszeit von Lehrkräften: Bestimmbar und unter Druck. Pädagogische Führung. (2018) 4:124–7.

[22] Gluschkoff K Elovainio M Kinnunen U Mullola S Hintsanen M Keltikangas-Järvinen L. Work stress, poor recovery and burnout in teachers. Occup Med. (2016) 66:564–70. doi: 10.1093/occmed/kqw086, PMID: 27412428

[23] Seibt R Spitzer S Blank M Scheuch K. Predictors of work ability in occupations with psychological stress. J Public Health. (2009) 17:9–18. doi: 10.1007/s10389-008-0194-9

[24] Seibt R Kreuzfeld S. Influence of work-related and personal characteristics on the burnout risk among full- and part-time teachers. Int J Environ Res Public Health. (2021) 18:1535. doi: 10.3390/ijerph18041535, PMID: 33562788 PMC7914652

[25] Varol YZ Weiher GM Wendsche J Lohmann-Haislah A. Difficulties detaching psychologically from work among German teachers: prevalence, risk factors and health outcomes within a cross-sectional and national representative employee survey. BMC Public Health. (2021) 21:2046. doi: 10.1186/s12889-021-12118-4, PMID: 34753459 PMC8576900

[26] Sonnentag S Fritz C. Recovery from job stress: the stressor-detachment model as an integrative framework. J Organ Behav. (2015) 36:S72–S103. doi: 10.1002/job.1924

[27] Cropley M Rydstedt LW Devereux JJ Middleton B. The relationship between work-related rumination and evening and morning salivary cortisol secretion. Stress Health. (2015) 31:150–7. doi: 10.1002/smi.2538, PMID: 24166947

[28] Guerts SAE Sonnentag S. Recovery as an explanatory mechanism in the relation between acute stress reactions and chronic health impairment. Scand J Work Environ Health. (2006) 32:482–92. doi: 10.5271/sjweh.1053, PMID: 17173204

[29] Vahle Hinz T Bamberg E Friedrich N Keller M. Effects of work stress on work-related rumination, restful sleep, and nocturnal heart rate variability experienced on workdays and weekends. J Occup Health Psychol. (2014) 19:217–30. doi: 10.1037/a0036009, PMID: 24635734

[30] Schulz AD Wendsche J Lohmann-Haislah A Schöllgen I. Erholungsbeeinträchtigungen bei Beschäftigten. Zbl Arbeitsmed. (2020) 70:57–65. doi: 10.1007/s40664-019-00373-7

[31] Bennett AA Bakker AB Field JG. Recovery from work-related effort: a meta-analysis. J Organ Behav. (2018) 39:262–75. doi: 10.1002/job.2217

[32] Wendsche J Lohmann-Haislah A. A meta-analysis on antecedents and outcomes of detachment from work. Front Psychol. (2017) 7:2072. doi: 10.3389/fpsyg.2016.02072 28133454 PMC5233687

[33] Rivkin W Diestel S Schmidt K-H. Psychological detachment: a moderator in the relationship of self-control demands and job strain. Eur J Work Organ Psychol. (2015) 24:376–88. doi: 10.1080/1359432X.2014.924926

[34] Xanthopoulou D Bakker AB Oerlemans WGM Koszucka M. Need for recovery after emotional labor: differential effects of daily deep and surface acting. J Organ Behav. (2018) 39:481–94. doi: 10.1002/job.2245

[35] Stults-Kolehmainen MA Sinha R. The effects of stress on physical activity and exercise. Sports Med. (2014) 44:81–121. doi: 10.1007/s40279-013-0090-5, PMID: 24030837 PMC3894304

[36] Litwiller B Snyder LA Taylor WD Steele LM. The relationship between sleep and work: a meta-analysis. J Appl Psychol. (2017) 102:682–99. doi: 10.1037/apl0000169, PMID: 27893255

[37] Sonnentag S. The recovery paradox: portraying the complex interplay between job stressors, lack of recovery, and poor well-being. Res Organ Behav. (2018) 38:169–85. doi: 10.1016/j.riob.2018.11.002

[38] Guseva Canu I Marca SC Dell'Oro F Balázs Á Bergamaschi E Besse C. Harmonized definition of occupational burnout: a systematic review, semantic analysis, and Delphi consensus in 29 countries. Scand J Work Environ Health. (2021) 47:95–107. doi: 10.5271/sjweh.3935, PMID: 33258478 PMC8114565

[39] Maslach C Leiter MP. Understanding the burnout experience: recent research and its implications for psychiatry. World Psychiatr. (2016) 15:103–11. doi: 10.1002/wps.20311, PMID: 27265691 PMC4911781

[40] Arvidsson I Leo U Larsson A Håkansson C Persson R Björk J. Burnout among school teachers: quantitative and qualitative results from a follow-up study in southern Sweden. BMC Public Health. (2019) 19:655. doi: 10.1186/s12889-019-6972-1, PMID: 31142318 PMC6542045

[41] Hultell D Gustavsson JP. Factors affecting burnout and work engagement in teachers when entering employment. Work. (2011) 40:85–98. doi: 10.3233/WOR-2011-1209, PMID: 21849751

[42] Scheuch K Haufe E Seibt R. Teachers' Health. Dtsch Arztebl Int. (2015) 112:347–56. doi: 10.3238/arztebl.2015.0347 26051692 PMC4558646

[43] Teles R Valle A Rodríguez S Piñeiro I Regueiro B. Perceived stress and indicators of burnout in teachers at portuguese higher education institutions. Int J Environ Res Public Health. (2020) 17:3248. doi: 10.3390/ijerph17093248, PMID: 32392696 PMC7246578

[44] Alarcon GM. A meta-analysis of burnout with job demands, resources, and attitudes. J Vocat Behav. (2011) 79:549–62. doi: 10.1016/j.jvb.2011.03.007

[45] Leung DYP Lee WWS. Predicting intention to quit among Chinese teachers: differential predictability of the components of burnout. Anxiety Stress Coping. (2006) 19:129–41. doi: 10.1080/10615800600565476

[46] Skaalvik EM Skaalvik S. Teacher job satisfaction and motivation to leave the teaching profession: relations with school context, feeling of belonging, and emotional exhaustion. Teach Teach Educ. (2011) 27:1029–38. doi: 10.1016/j.tate.2011.04.001

[47] Van Droogenbroeck FV Spruyt B. To stop or not to stop: an empirical assessment of the determinants of early retirement among active and retired senior teachers. Res Aging. (2014) 36:753–77. doi: 10.1177/0164027513519449 25651547

[48] Statistisches Bundesamt. (2018). Zahl der Pensionierungen von Lehrkräften 2017 erneut rückläufig. Available at: https://www.destatis.de/DE/Presse/Pressemitteilungen/2018/12/PD18_509_742.html;jsessionid=A60AFCBFAA01AE32EFB461938EA17717 (Accessed August 09, 2023)

[49] Kreuzfeld S Seibt R. Gender-specific aspects of teachers regarding working behavior and early retirement. Front Psychol. (2022) 13:829333. doi: 10.3389/fpsyg.2022.829333, PMID: 35242087 PMC8887565

[50] Voglino G Savatteri A Gualano MR Catozzi D Rousset S Boietti E. How the reduction of working hours could influence health outcomes: a systematic review of published studies. BMJ Open. (2022) 12:e051131. doi: 10.1136/bmjopen-2021-051131, PMID: 35365508 PMC8977802

[51] Richter P Rotheiler E Rudolf M. FABA – Fragebogen zur Analyse belastungsrelevanter Anforderungsbewältigung. Mattersburg: P & T Prieler Tometich (2015).

[52] Richter P Rudolf M Schmidt CF. FABA: Fragebogen zur Erfassung beanspruchungsrelevanter Anforderungsbewältigung. Frankfurt/M: Harcourt Test Service (1996).

[53] Blanz M. Gütekriterien von Testverfahren In: Blanz M, editor. Forschungsmethoden und Statistik für die Soziale Arbeit. Grundlagen und Anwendungen. Stuttgart: Kohlhammer (2015). 255–9.

[54] Schaufeli WB Leiter MP Maslach C Jackson SE. Maslach burnout inventory – general survey In: Maslach C Jackson SE Leiter MP, editors. Maslach burnout inventory – manual. Palo Alto, CA: Consulting Psychologists Press (1996). 19–26.

[55] Kalimo R Pahkin K Mutanen P Topipinen-Tanner S. Staying well or burning out at work: work characteristics and personal resources as long-term predictors. Work Stress. (2003) 17:109–22. doi: 10.1080/0267837031000149919

[56] Mayring P. Qualitative Inhaltsanalyse In: Mey G Mruck K, editors. Handbuch Qualitative Forschung in der Psychologie. Wiesbaden: Springer (2010). 601–13.

[57] Bortz J Schuster C. Statistik für Human- und Sozialwissenschaftler. Heidelberg: Springer Medizin Verlag (2010). p. 153–182.

[58] Nagelkerke NJD. A note on a general definition of the coefficient of determination. Biometrika. (1991) 78:691–2. doi: 10.1093/BIOMET/78.3.691

[59] Cohen J. Statistical power analysis for the behavioral sciences. Hillsdale, NJ: Lawrence Erlbaum Associates (1988).

[60] Lenhard W Lenhard A. (2015) Berechnung von Effektstärken. Available at: https://www.psychometrica.de/effektstaerke.html (Accessed April 17, 2023)

[61] Mußmann F Hardwig T Haunschild A. Berechnung und Gestaltung der Arbeitszeit von Lehrkräften – Entwicklung arbeitszeitrechtlicher Normen für Lehrerinnen und Lehrer sowie Schulleitungen. Recht der Jugend und des Bildungswesens. (2019) 67:298–311. doi: 10.5771/0034-1312-2019-3-298

[62] Backhaus K Erichson B Gensler S Weiber R Weiber T. Logistische Regression In: Backhaus K Erichson B Gensler S Weiber R Weiber T, editors. Multivariate Analysemethoden: Eine anwendungsorientierte Einführung. Wiesbaden: Springer Fachmedien (2021). 289–382.

[63] Chen H Cohen P Chen S. How big is a big odds ratio? Interpreting the magnitudes of odds ratios in epidemiological studies. Comm Stat Simul Comput. (2010) 39:860–4. doi: 10.1080/03610911003650383

[64] Sandmeier A Baeriswyl S Krause A Muehlhausen J. Work until you drop: effects of work overload, prolonging working hours, and autonomy need satisfaction on exhaustion in teachers. Teach Teach Educ. (2022) 118:103843. doi: 10.1016/j.tate.2022.103843

[65] Seibt R Matz A Hegewald J Spitzer S. Working conditions of female part-time and full-time teachers in relation to health status. Int Arch Occup Environ Health. (2012) 85:675–87. doi: 10.1007/s00420-011-0715-7, PMID: 22038088

[66] Mummert CA. (2005). Das Lehrerarbeitszeitmodell in Hamburg. Bericht zur Evaluation. Available at: https://www.hamburg.de/contentblob/70400/d0ac91b7862358862ec1cbd52864a741/data/laz-bericht.pdf (Accessed July 13, 2023)

[67] Sellen P. (2016). Teacher workload and professional development in England's secondary schools: Insights from TALIS. Available at: https://epi.org.uk/publications-and-research/teacher-workload-professional-development-englands-secondary-schools-insights-talis/ (Accessed April 17, 2023)

[68] Gicheva D. Altruism and burnout: long hours in the teaching profession. ILR Rev. (2020) 75:427–57. doi: 10.1177/0019793920981055

[69] Schiller H Lekander M Rajaleid K Hellgren C Åkerstedt T Barck-Holst P. Total workload and recovery in relation to worktime reduction: a randomised controlled intervention study with time-use data. Occup Environ Med. (2018) 75:218–26. doi: 10.1136/oemed-2017-104592, PMID: 29183947 PMC5869453

[70] Unterbrink T Hack A Pfeifer R Buhl-Grießhaber V Müller U Wesche H. Burnout and effort–reward-imbalance in a sample of 949 German teachers. Int Arch Occup Environ Health. (2007) 80:433–41. doi: 10.1007/s00420-007-0169-0, PMID: 17294239

[71] Bodendieck E Jung FU Luppa M Riedel-Heller SG. Burnout and work-privacy conflict – are there differences between full-time and part-time physicians? BMC Health Serv Res. (2022) 22:1082. doi: 10.1186/s12913-022-08471-8, PMID: 36002851 PMC9404597

[72] Du Bois K Sterkens P Lippens L Baert S Derous E. Beyond the hype: (how) are work regimes associated with job burnout? Int J Environ Res Public Health. (2023) 20:3331. doi: 10.3390/ijerph20043331, PMID: 36834026 PMC9965496

[73] Buhl J Acosta J. Work less, do less? Sustain Sci. (2016) 11:261–76. doi: 10.1007/s11625-015-0322-8

[74] Neubert S Bader C Hanbury H Moser S. Free days for future? Longitudinal effects of working time reductions on individual well-being and environmental behaviour. J Environ Psychol. (2022) 82:101849. doi: 10.1016/j.jenvp.2022.101849

[75] Wang Y Ramos A Wu H Liu L Yang X Wang J. Relationship between occupational stress and burnout among Chinese teachers: a cross-sectional survey in Liaoning, China. Int Arch Occup Environ Health. (2015) 88:589–97. doi: 10.1007/s00420-014-0987-9 25256806

[76] Gyllensten K Palmer S. The role of gender in workplace stress: a critical literature review. Health Educ J. (2005) 64:271–88. doi: 10.1177/001789690506400307

[77] Eurofound. (2017). Sixth European Working Conditions Survey – Overview report (2017 update). Available at: https://www.eurofound.europa.eu/publications/report/2016/working-conditions/sixth-european-working-conditions-survey-overview-report (Accessed July 13, 2023)

[78] Ptáček R Vnukova M Raboch J Smetackova I Sanders E Svandova L. Burnout syndrome and lifestyle among primary school teachers: a Czech representative study. Med Sci Monit. (2019) 25:4974–81. doi: 10.12659/MSM.914205, PMID: 31274132 PMC6626498

[79] Harris DN Adams SJ. Understanding the level and causes of teacher turnover: a comparison with other professions. Econ Educ Rev. (2007) 26:325–37. doi: 10.1016/j.econedurev.2005.09.007

[80] Bakker AB de Vries JD. Job demands–resources theory and self-regulation: new explanations and remedies for job burnout. Anxiety Stress Coping. (2021) 1:1–21. doi: 10.1080/10615806.2020.1797695 32856957

[81] Babic S Mairitsch A Mercer S Sulis G Jin J King J. Late-career language teachers in Austria and the UK: pathways to retirement. Teach Teach Educ. (2022) 113:103686. doi: 10.1016/j.tate.2022.103686

[82] Fisher GG Chaffee DS Sonnega A. Retirement timing: a review and recommendations for future research. Work Aging Retire. (2016) 2:230–61. doi: 10.1093/workar/waw001

[83] Topa G Depolo M Alcover C-M. Early retirement: a meta-analysis of its antecedent and subsequent correlates. Front Psychol. (2018) 8:2157. doi: 10.3389/fpsyg.2017.02157, PMID: 29354075 PMC5759094

[84] Organization for Economic Co-operation and Development. (2005). Teachers matter: attracting, developing and retaining effective teachers. Available at: https://www.oecd.org/education/school/34990905.pdf (Accessed July 13, 2023)

